# Supplementary material for: A multifunctional soft robotic shape display with high-speed actuation, sensing, and control
Source: Nat Commun. 2023 Jul 31;14:4516. doi: 10.1038/s41467-023-39842-2 (PMC10390478; doi:10.1038/s41467-023-39842-2)
Supplement: Supplementary file 1 — Supplementary Information [file 41467_2023_39842_MOESM1_ESM.pdf]

Supplementary Information for

A multifunctional soft robotic shape display with high-speed actuation, sensing, and control

B.K. Johnson[1]†, M. Naris[1]†, V. Sundaram[1], A. Volchko[1], K. Ly[1], S.K. Mitchell[1,2], E. Acome[1,2], N. Kellaris[1,2,3], C. Keplinger*[1,3,4], N. Correll*[5], J.S. Humbert*[1], M.E. Rentschler*[1]

[1]Paul M. Rady Mechanical Engineering, University of Colorado Boulder; Boulder, USA.
[2]Artimus Robotics; Boulder, USA.
[3]Materials Science and Engineering Program, University of Colorado Boulder; Boulder, USA
[4]Robotic Materials Department, Max Planck Institute for Intelligent Systems; Stuttgart, Germany.
[5]Department of Computer Science, University of Colorado Boulder; Boulder, USA.

*Correspondence to: mark.rentschler@colorado.edu, sean.humbert@colorado.edu, nikolaus.correll@colorado.edu, ck@is.mpg.de
†These authors contributed equally to this work.

Contents:

Supplementary Figures
Supplementary Figure 1: HV driver circuit schematic
Supplementary Figure 2: HASEL actuator charge/discharge rates
Supplementary Figure 3: Motion capture camera setup
Supplementary Figure 4: Force sensing resolution
Supplementary Figure 5: Magnetometer and SPI topology
Supplementary Figure 6: Module driver circuit schematic
Supplementary Figure 7: Module sensor circuit schematic
Supplementary Figure 8: Power distributor circuit schematic for a single module
Supplementary Tables
Supplementary Table 1: Parameter values for force estimation
Supplementary Table 2: 10x10 shape display component breakdown
Supplementary Methods

Supplementary Figures:

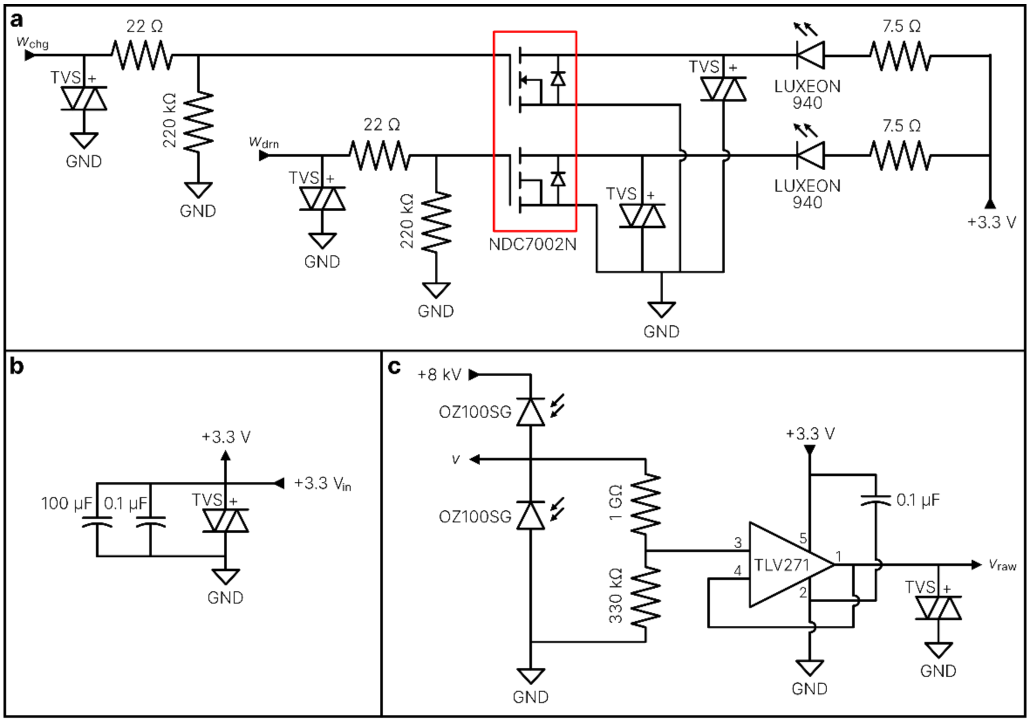

Supplementary Figure 1: HV driver circuit schematic. (**A**) The duty cycles of the infrared LEDs (LUXEON940) are controlled by the w_{chg} and w_{drn} input signals from the module driver circuit (Supplementary Fig. 6). Transient voltage suppressor (TVS) diodes act as a safety measure to drain charge in the event of electrostatic discharge. (**B**) A TVS diode and buffer capacitors also connect the input voltage to ground (GND). (**C**) The HASEL actuator voltage v is controlled by the two HV diodes (OZ100SG). The 8 kV input comes from the module HV power supply. An op-amp-driven voltage divider acts as a HV sensor, providing a low voltage signal v_{raw} to the module driver circuit.

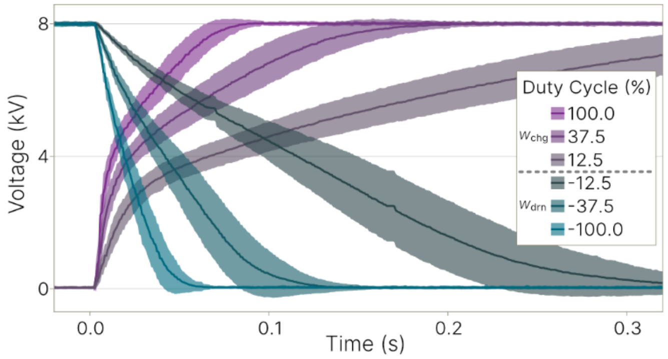

Supplementary Figure 2: HASEL actuator charge/discharge rates for selected duty cycles (%) across 100 actuators on the surface. Shaded areas represent +/- 1 standard deviation; solid lines are the mean value, n = 100.

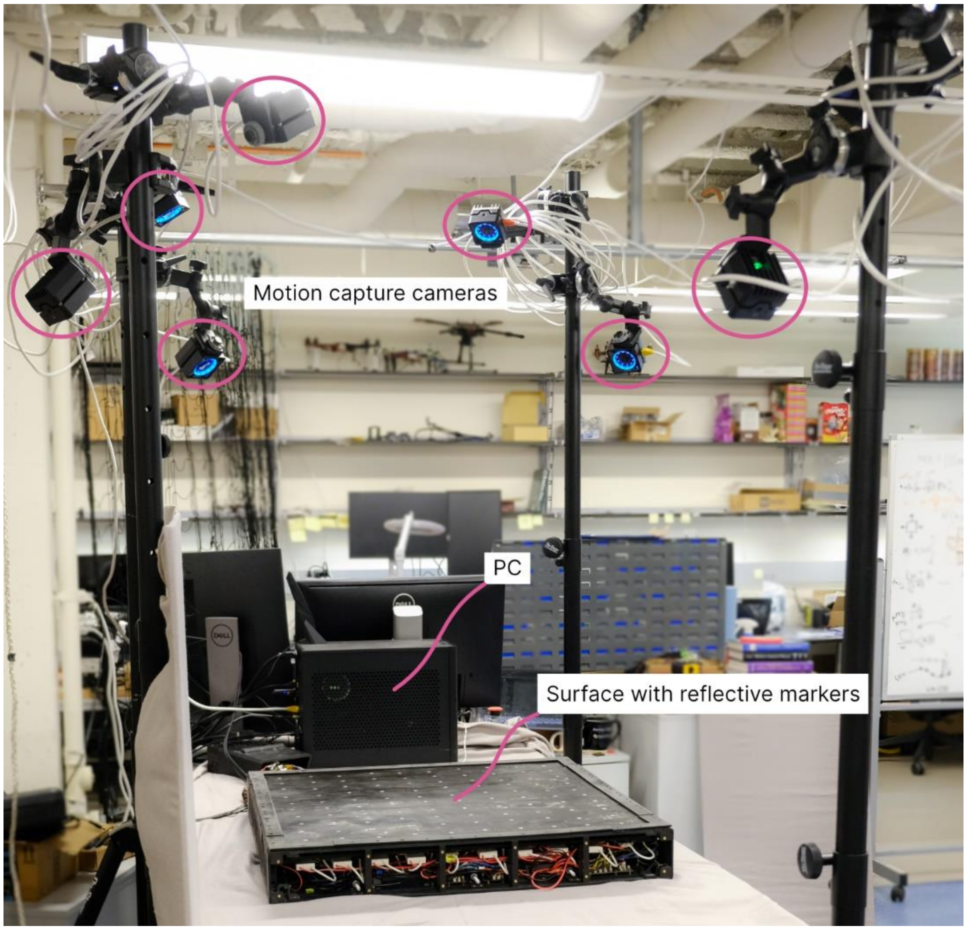

Supplementary Figure 3. **Motion capture camera setup.** The cameras register the position of the 100 reflective markers (one at the center of each pixel cell). The PC streams the motion capture data via ethernet connection at 240 Hz.

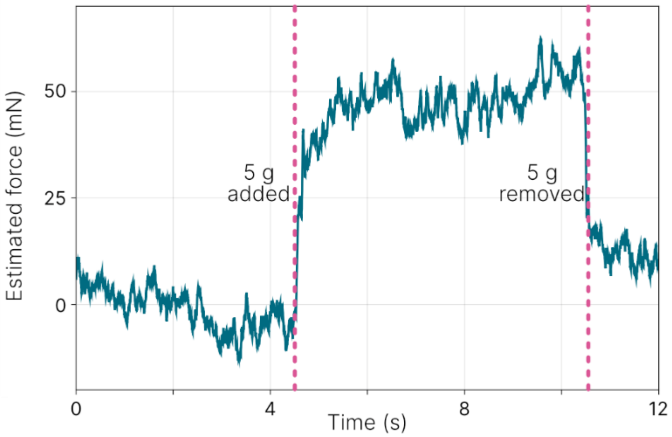

Supplementary Figure 4: Force sensing resolution. Example of a single cell detecting the change in force when a 5 g mass (50 mN force) is added on top of the cell.

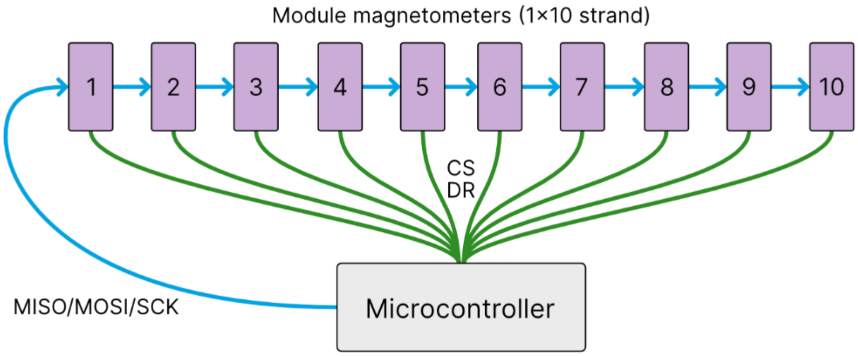

Supplementary Figure 5: Magnetometer and SPI topology. The magnetometers are wired in series within each 1x10 module. The MISO (Master In Slave Out), MOSI (Master Out Slave In), and SCK (Clock) signals are daisy chained between magnetometers; individual CS (Chip Select) and DR (Data Ready) signals run from each magnetometer to the module sensor circuit.

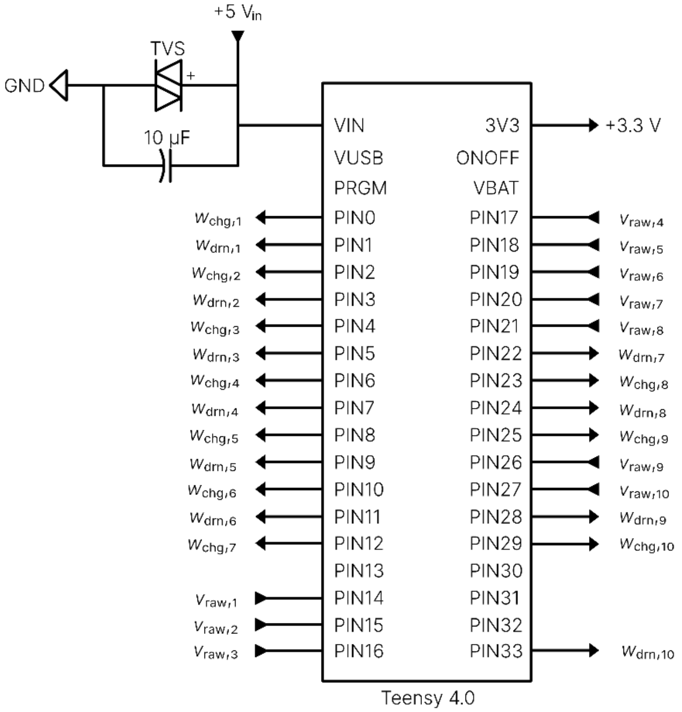

Supplementary Figure 6: Module driver circuit schematic. Each HV driver in the module (1-10) receives the corresponding w_{chg} and w_{drn} signal (Supplementary Fig. 1a). From each HV driver, the module driver circuit receives the corresponding v_{raw} signal (Supplementary Fig. 1c).

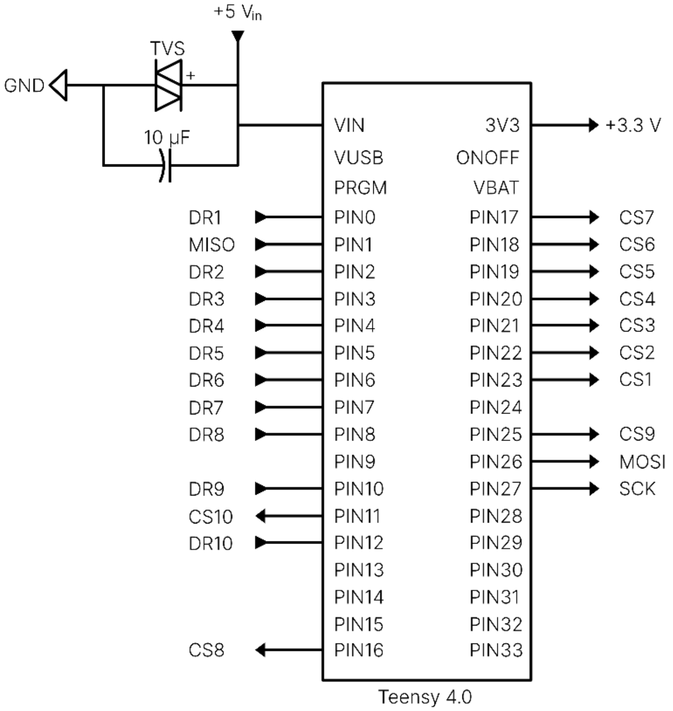

Supplementary Figure 7: Module sensor circuit schematic. The common lines MISO, MOSI, SCK are connected in series through the magnetometers in the module (Supplementary Fig. 5). Each magnetometer (#1-10) connects to the corresponding CS and DR pins.

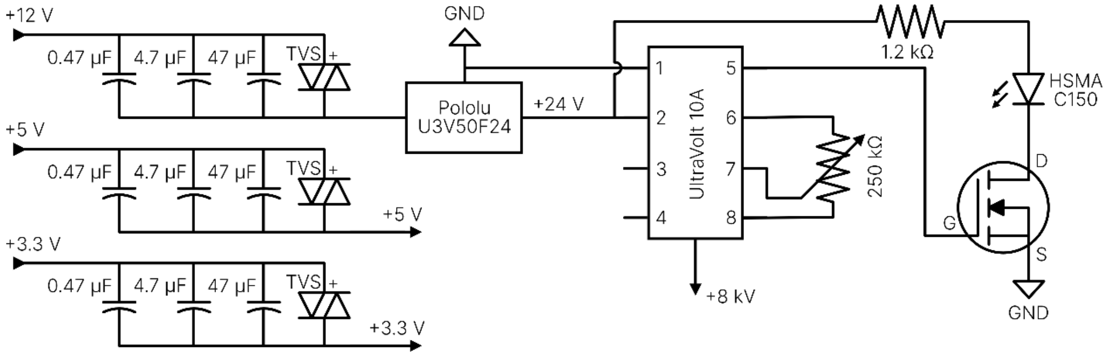

Supplementary Figure 8: Power distributor circuit schematic for a single module. The AC/DC power supply provides +12 V, +5 V, and +3.3 V inputs to the circuit. The +3.3 V and +5 V rails are supplied to the HV drivers, module driver circuit, and module sensor in the module. The +12 V signal is amplified (Pololu U3V50F24) to +24 V to supply the HV power supply (UltraVolt 10A), which supplies +8 kV to the HV drivers. An LED (HSMA C150) provides visual feedback: if the LED is lit, HV power is being supplied to the system.

Supplementary Tables:

Supplementary Table 1. Parameter values for force estimation

Parameter	Value
p_0	1.854 x 10^{-1}
p_1	-6.493 x 10^{-2}
p_2	2.380 x 10^{-1}
p_3	4.649 x 10^{-3}
p_4	-9.134 x 10^{-2}
p_5	8.367 x 10^{-3}
p_6	7.572 x 10^{-3}
p_7	5.886 x 10^{-4}
p_8	3.042 x 10^{-4}
p_9	-8.167 x 10^{-5}
p_{10}	-2.290 x 10^{-5}
p_{11}	-5.642 x 10^{-5}
p_{12}	-1.710 x 10^{-5}
p_{13}	-1.266 x 10^{-5}
p_{14}	-7.245 x 10^{-6}

Supplementary Table 2. 10x10 shape display component breakdown

Component	Quantity	Source/Subcomponents	Approx. cost per unit (USD)
HASEL actuator	100	See Supplementary Methods	0.10 (42)
Magnetic block	100	See Supplementary Methods	N/A
Magnetic sensor strand	10	See Supplementary Methods	100
	100	LIS3MDL Carrier, Pololu	
HV driver board	100	See Supplementary Methods	100
	100	Custom PCB, PCBWay	
	200	OZ100SG, Voltage Multipliers, Inc.	
HV power supply	10	UltraVolt 10A24-P30, Advanced Energy	750
Microcontroller	20	Teensy 4.0, PJRC	20
Module 24 V amplifier	10	Pololu U3V50F24	35
Module PCBs	30	Custom PCBs, PCBWay	20
AC/DC power supply	1	ION SFX 650G, Fractal Design	150

Supplementary Methods:

HASEL fabrication

The method of creating the folded HASEL actuators in this work follows the fabrication method of Mitchell *et al.* (46). Each folded HASEL is formed from a continuous film of 24 actuator shells. The film is folded with 12 accordion folds leaving two shells per layer. We form the film using two layers of 20 μm thick polyester lidding film (L0WS, Multi-Plastics) heat-sealed together with a modified CNC machine (Shapeoko 3XL, Carbide 3D). The CNC machine seals the individual shells at 195 °C. We then deposit a thin flexible layer of conductive ink (CI-2051, Engineered Materials System) on both sides of the sealed film using a screen-printing process. Each actuator shell is filled with 0.4 mL silicone liquid dielectric (PSF-5cSt, Clearco) through a syringe injection into the film. The silicone dielectric is a low viscosity fluid which improves the actuator's dynamic performance. Any large bubbles (above 3 mm diameter) are forced out of the pouch before the fill port is sealed with a soldering iron. Once the line of shells is complete, thin strips of transfer tape (924, 3M) are used to secure the accordion fold. Lastly, a rigid circuit board is mounted to the actuator film using double-sided tape (Scotch, 3M); Conductive Carbon Glue (Pelco) is used to form a conductive bond between the film electrodes and the circuit board. The mounted board provides a conductive interface that mounts to the 1x10 module hardware, connecting the HASEL actuator to the HV electronics. This process is repeated for all 100 actuators in the shape display.

Magnetic block fabrication

We based the fabrication process of the magnetic silicone blocks on Sundaram *et al.* (36). The magnetic components of the sensing mechanism are flexible, silicone blocks (EcoFlex 00-30, Smooth On) suffused with bonded neo-powder (NQP-B+ 20441, Neo

Magnequench). First, we hand-mixed EcoFlex 00-30 Part A and Part B rubbers using a 1:1 weight ratio. We then added and hand-mixed in 1.67 weight % of neo-powder. Once most of the powder was roughly distributed in the silicone, we placed the mixture into a planetary mixer (ARV-310, Thinky). The mixture was first degassed in a vacuum (~0.2 kPa) for 60 s, then mixed at a speed of 2000 rpm for 30 s, before the speed decreased to 200 rpm at 10.5 kPa for 60 s.

We poured the mixture into a 42 mm x 42 mm x 4 mm acrylic mold and placed it in the center of the empty volume between two ND42 10.16 cm x 10.16 cm x 2.54 cm magnets (CMS Magnetics) separated by 4 cm. The mixture was cured for about 2.5 hr before being removed from the acrylic mold. This process polarizes the magnetic particles in the mixture as the silicone cures. The process was repeated for all 100 magnetic blocks in the shape display.

Magnetic sensing circuit and system assembly

A low cost, off-the-shelf 3-axis magnetometer (LIS3MDL, ST Electronics) on a breakout board (LIS3MDL Carrier, Pololu) was used for the magnetometer circuit. The module-level sensor microcontroller (Teensy 4.0, PJRC) receives raw magnetic flux density data from the magnetometers via a four-wire SPI (serial peripheral interface) communication protocol (Supplementary Fig. 5). The SPI clock speed was set to 1 MHz with SPI Mode 0. Since we only used the magnetic flux density measurement in one-dimension, no additional calibration to account for external magnetic sources is required.

Magnetic force curve fit

The force sensing experiments were performed as described in the Materials and Methods. Using the MATLAB Curve Fitting toolbox (MathWorks), the data was used to generate a 15th-order polynomial fit which maps from the measured voltage $\hat{v}$ and displacement $\hat{z}$ to an estimated force $\hat{f}$:

$$\hat{f} = p_0 + p_1\hat{v} + p_2\hat{z} + p_3\hat{v}^2 + p_4\hat{v}\hat{z} + p_5\hat{z}^2 + p_6\hat{v}^2\hat{z} + p_7\hat{v}\hat{z}^2 + p_8\hat{z}^3 + p_9\hat{v}^2\hat{z}^2 + p_{10}\hat{v}\hat{z}^3 + p_{11}\hat{z}^4 + p_{12}\hat{v}^2\hat{z}^3 + p_{13}\hat{v}\hat{z}^4 + p_{14}\hat{z}^5. \quad (1)$$

The parameter values for Supplementary equation (1) are listed in Supplementary Table 1.

HV driver fabrication

The HV driver (Supplementary Fig. 1) was commercially fabricated (PCBWay) with all surface-mount components soldered at the factory. The remaining fabrication was done by hand. Each optocoupler in the circuit consists of a low voltage infrared LED (L1IZ-0940000000000, Lumileds) and an HV photodiode (OZ100SG, Voltage Multipliers, Inc.). To consistently align the LEDs with the photodiodes during assembly, we milled a 1350 x 2750 µm slot into each photodiode which fits over the surface-mounted LED. The two photodiodes and an HV resistor for the HV sensor were hand-soldered onto the driver board. Wires to connect the board to the HASEL actuator electrodes and to GND were also added.

We potted the HV components in an insulating epoxy. Between the photodiode and LED, we used a transparent epoxy (Quickset Clear, Pratley) to not impede the function of the

LED. After curing, we encased the remaining HV components in opaque epoxy (Pratley White Epoxy, Pratley).

Optocoupler charge rate quantification

To measure the charge rate of the HV driver in each cell (Fig. 2d), we input a set of 16 PWM duty cycles for both w_{chg} and w_{drn}, linearly spaced from 0 – 100 %. All 100 cells received the inputs simultaneously. For each duty cycle, we recorded the cell voltage as a function of time across all 100 cells. The charge rate (kV s^{-1}) was manually derived by taking the 10-90% voltage differential (6.4 kV) and dividing by the measured 10-90% rise time. The resulting data is shown in Fig. 2d. Additional charge/discharge time data for select duty cycles is shown in Supplementary Fig. 2.

Shape display 10-by-10 assembly

The full 10x10 shape display is assembled by first assembling 10 identical modules. Each module is constructed from a series of structural components which integrate the various cell and module electronics. The structure of each module consists of an acetal base and top plate plus 3D-printed (ColorFabb XT-CF20, Prusa MK3S) support pillars. Acetal front and side panels are also added to enclose the space. The unactuated footprint of each cell (including the acetal hardware) is 6 cm x 6 cm with a 9 cm height. A 13.2 nF capacitor bank is created for each module by soldering four 3.3 nF capacitors in parallel. The HV power supply (UltraVolt 10A24-P30, Advanced Energy), capacitor bank, module driver circuit, module sensor circuit, power distributor board, and other components are placed on the base plate (Fig. 1c) and secured with screws. The capacitor bank electrodes are connected to the HV power supply power and ground lines. The 10 driver boards and magnetic sensor boards for each cell are screwed to the underside of the top plate. Power

and communication cables are routed through the module cavity. The module-level driver circuit, sensor circuit, and power distributor boards (Supplementary Figs. 6, 7, 8) were fabricated commercially (PCBWay). All electrical components were soldered by hand onto the boards.

The 1x10 array modules are horizontally stacked next to each other to form the 10x10 array. On the left and right side (the 1st and 10th module), a passive end support structure is added which helps secure the silicone skin on the top of the display. Before full assembly, the power and ground cables are run perpendicular to each module and exit out a hole on one of the end support structures. USB cables from each module driver and module sensor circuits are collected into USB hubs for a total output of 2 USB cables. The cables are also run out the hole in the end support structure towards the PC. The 10 modules are secured with threaded rods and nuts at the front, center, and back of each module. The power cables are plugged into a custom board which interfaces with the AC/DC power supply (ION SFX 650G, Fractal Design). The board connects the 3.3 V, 5 V, and 12 V wiring from the modules to the respective AC/DC outputs. For additional safety grounding, the ground rail of each module (from the HV power supply) is run outside of the device and connected directly to the building ground.

The silicone skin is fabricated by mixing 100 g part A and 100 g part B of EcoFlex 00-30 (Smooth On) with 3% by weight black Silc-Pig (Smooth On). The mixture is poured into a square frame mold on a tabletop with an area of 62 cm x 62 cm and left to settle to an average thickness of 550 μm. To provide a matte smooth surface, we mix equal parts A and B of Psycho Paint (Smooth On), add 400% by weight of solvent (NOVOCS Matte, Smooth On), 3% by weight Silc-Pig, and spread the mixture evenly over the cured

EcoFlex 00-30 surface. After drying, the silicone skin is peeled off the table and carefully stretched over the 10x10 display. Panels secured by screws along each edge of the array are used to hold the skin in place.

A component and cost breakdown of major components of the display is shown in Supplementary Table 2. The largest costs at the module level are the HV power supply and the individual HV driver circuit for each actuator (primarily due to the cost of HV photodiode). We expect in the future that switching to a MOSFET-based half bridge in the driver circuit would significantly reduce cost and power consumption of the circuit.

Software and firmware

Both the module driver circuit and module sensor circuit use a Cortex-M7 based microcontroller (Teensy 4.0, PJRC), programmed using the Arduino framework. Each microcontroller connects to the central PC over USB 2.0 as a HID device to ensure bounded latency communication.

We implemented the central communication and control code in Julia 1.7 (59) running on Ubuntu 21.04 (Canonical, Linux Kernel 5.11). The code was divided between multiple functional units with information shared between each. Individual functional units, such as control laws, run as asynchronous tasks distributed across all 16 threads of the CPU (Ryzen 5800X, Advanced Micro Devices). Each task is scheduled at a set frequency (for example, sensor data sampling runs at 600 Hz, while deformation control loops run at 200 Hz). Data is shared between tasks in a loss-tolerant, UDP-like manner using thread-safe shared memory. Data was processed with the following Julia packages: JLD2, GeometryBasics, ScatteredInterpolation, Statistics, FFTW, DifferentialEquations, DSP,

LinearAlgebra, Polynomials, and Loess. Visualizations were rendered using Makie.jl (60). For the ball manipulation experiments, Python and OpenCV (58) were used to generate the ball position information from the USB camera.

The software is available as Supplementary Software 1.
