# Supplementary material for: A multifunctional soft robotic shape display with high-speed actuation, sensing, and control
Source: Nat Commun. 2023 Jul 31;14:4516. doi: 10.1038/s41467-023-39842-2 (PMC10390478; doi:10.1038/s41467-023-39842-2)
Supplement: Supplementary file 3 — Description of Additional Supplementary Files [file 41467_2023_39842_MOESM3_ESM.pdf]

Description of Additional Supplementary Files

Supplementary Movie 1: Overview of capabilities. We present a variety of demonstrations and capabilities shown by the soft robotic shape display.

Supplementary Movie 2: Noise level of the soft surface (100 cells). Actuation of all 100 cells of the surface at peak operation (12 Hz motion) results in an 8 dB increase of noise over ambient room conditions. Slower operations result in only a brief 1-2 dB noise level increase.

Supplementary Movie 3: Real-time displacement map of an actuated surface ripple. The shape display demonstrates fluidic motion such as this example which creates a three-dimensional ripple effect. Using the embedded magnetic sensors, it is possible to generate a real-time displacement map of the ripple.

Supplementary Movie 4: Scrolling text display application. Here, we demonstrate an application of the soft surface by scrolling text ("HELLO") across the surface.

Supplementary Movie 5: Surface stimulus reaction through self-sensing. One half of the display acts as a passive deformation sensor. When a user presses on the passive surface, the other half of the display actuates corresponding to the position and amplitude of the press.

Supplementary Movie 6: Interactive scale with text display. By combining force sensing and text display capabilities, the surface is able to form a smart scale that can sense object mass and display the resulting value.

Supplementary Movie 7: Simultaneous multifunctionality of the soft surface. The soft shape display simultaneously performs multiple functions in different spatial regions of the surface.

Supplementary Movie 8: User interaction by magnetic wand drawing. Using a wand with a hard magnetic tip that interacts with the magnetometers inside the display, the shape display demonstrates active user applications like real-time surface drawing.

Supplementary Movie 9: Closed-loop object manipulation – rolling a ball in a square trajectory. A table tennis ball is pushed across the surface using feedback-control driven surface morphology changes through a set of goal positions, forming a square trajectory.

Supplementary Movie 10: Closed-loop object manipulation – sorting three balls by color detection. Three table tennis balls of different colors are sorted to different goal regions based on ball color. Ball motion is driven by surface morphology changes.

Supplementary Software 1: Full scripts used for microcontroller closed loop control, PC closed loop control and communication, motion capture data processing, and test data collecting and processing.
